# Neurodiversity studies: mapping out possibilities of a new critical paradigm

**DOI:** 10.1080/09687599.2021.1919503

**Published:** 2021-06-24

**Authors:** Anna Stenning, Hanna Bertilsdotter-Rosqvist

**Affiliations:** University of Leeds, UK; Södertörn University, Sweden

## Abstract

Neurodevelopmental classifications and the collective idea of neurodivergence can be seen as a ‘moving target’. In our understanding, this means that it responds to the needs of society as well as potentially infinite neurological differences between humans. Therefore, rather than assume that neurodiversity exists according to the existing clinical categories of autism and related conditions (that are often centred around autism as the exemplary kind of neurodivergence), we leave the possibility open that there are other forms of difference that have yet to be defined. In the paper we explore how neurodiversity has been described as a collective property of brains, as we try to negotiate between us what it is to be human and how we can work together to ensure our flourishing and to alleviate suffering. We consider implications of this understanding of neurodiversity for autism research, and propose that we unpick the analogy between neurodiversity and biodiversity.

Neurodiversity is an idea that is much talked about and it has several meanings. In its most common usage, neurodiversity is used to refer to a supposedly natural variation in the ‘kinds’ of brains in developmental terms akin to biological diversity, which is used to describe the variety of living (nonhuman) creatures (Wilson, 1985). It is assumed that these brains – labelled as impaired and autistic, ADHD, dyslexic or Tourette’s by psychologists and as ‘neurodivergent’ by activists – conform to categories that reflect the underlying structure of reality as ‘natural kinds’, which will eventually be confirmed by neuroscientists. Like biodiversity, the term neurodiversity has come to be used for different purposes, but most often with an assumption that what is natural is always beneficial, which is at odds with the purpose of clinical research that seeks to alleviate suffering. The problem with this is that it both draws on the ideas of contemporary psychological science without critiquing their conceptual connotations whereby ‘cognitive differences’ are only understood in relation to falling short of an ideal cognitive type, and therefore it does not recognise difference as positive akin to the ecological ideal of biodiversity.

As Robert Chapman has pointed out in our edited book, Neurodiversity Studies: A New Critical Paradigm (Bertilsdotter Rosqvist et al, 2020), it is possible to understand the neurodevelopmental classifications and the collective idea of neurodivergence as a ‘moving target’ (Chapman 2020). In our understanding, this means that it responds to culture and society as well as potentially infinite neurological differences between humans. Therefore, rather than assume that neurodiversity exists according to the existing clinical categories of autism and related conditions that are discovered by impartial observers, we leave open the possibility that what counts as significant difference between brains should be reconsidered. This requires reconsidering what it means to be human in the light of contemporary ecological understandings that recognise interdependence rather than the hierarchical divisions of eugenics. We consider implications of this understanding of neurodiversity for autism research, and propose that we unpick the analogy between neurodiversity and biodiversity.

Back in the 1980s, the biologist E.O. Wilson argued for the need to quantify existing biological species for three different reasons: to discover the impact of human habitat destruction, to understand our ‘place in the order of things’ and, more practically, to determine the economic benefit that might be obtained from underexploited species. Neurodiversity was defined in the light of Judy Singer’s desire to give real credit to the ‘diverse bodies and minds’, which she believed had hitherto been overlooked by disability studies through its rejection of medical science, particularly in relation to the categories of Asperger’s Syndrome or High Functioning Autism (Singer 2017: Kindle ref 182). While she realised that focusing on material difference could give rise to ‘biological determinism’, her own position was akin to that of first-wave feminists seeking equality and representation based on differences that could be recognised at a biological level. For Singer, as for Harvey Blume with whom she shared common ground, neurodiversity like its ecological sciences analogy, also offered an opportunity for demonstrating the pragmatic value of high functioning autistic people in certain occupations (Blume 1998). However, celebrating Asperger’s Syndrome in terms of social usefulness reinforces the social Darwinist paradigm upon which cognitive psychology is typically based.

This coincided with contemporary work in clinical psychology that focused on discovering the diverse manifestations of autism, specifically in relation to those who were considered to have strengths in computing and technology. But Singer also hinted that she also intended to address other conditions that had formerly been described as mental illness. So, while initial work focused exclusively on Asperger’s Syndrome defined as a difference in sensory experience and processing (rather than impairments), she suggested that the idea might equally apply to other ‘properties of the mind’ (Singer 2017: location 178).

Comparable to Blume’s discussion of the potentially positive aspects of autism at the expense of difficulties, neurodiversity has been appropriated in employment, social welfare educational paradigms of best practice, where interventions aim at fitting ‘neurodivergent people’ into established hierarchies and ways of doing things. From this usage, and despite Singer’s advocacy for more diverse understandings, neurodiversity most often serves as a tool for othering neurodivergent students and employees. Within the neoliberal economy, it serves to support “NT business as usual” (Bertilsdotter Rosqvist et al, 2020), where the perspective of “majority” neurological type is continued to be taken for granted.

This parallels the issues that have been faced within ecology, between talk of ‘intrinsic value’ in non-human nature and estimations of commercial use-value. While neurodiversity may be described as ‘important for species survival’ (Singer 292: Blume 2008), this utilitarian inflection of neurodiversity may be responsible for the backlash against the idea that it ignores those who are outside of the economic marketplace. Furthermore, it suggests that neurodiversity is something that can be measured in a quantitative way.

We believe that neurodiversity should be understood from a third perspective. This third perspective stems principally from ideas of ‘autistic self-advocacy’ and online communities for the ‘neurodivergent’, queer or Mad. This perspective focuses, to paraphrase Jim Sinclair’s language, on ‘problems that neurodivergent people have’ rather than the ‘problem we are’ (1992). While this conception of neurodiversity originates in medical diagnostic procedures, its authority should not depend on clinical recognition or ‘scientific engines of discovery’ (Hacking 2007), and it allows for the possibility of self-diagnosis as equally valid.

Our edited book suggests a move beyond these positions, developed on the ‘shoulders’ of the third perspective. We offer a methodology that may be incorporated in various academic disciplines, but also which suggests the importance of a change in perspective: from “NT business as usual” to greater recognition, representation and resources for those at the neurological margins of academic discourse.

We therefore question the association of neurodiversity with strengths that benefit humankind in general, and with ‘natural’ properties of individual humans. If we talk instead about neurodivergent ‘ways of being’ (Sinclair 1992) we discover that the benefits of particular neurological conditions are dependent on the social practices and institutions that exist at a particular time. Investigations by psychologists and geneticists founded on the idea that autism exists as a monolithic ‘natural kind’ of human being has failed to reveal any useful knowledge in the past 80 years of research. Instead, we find little snippets of information about what autism is not.

For instance, what is thought of as being a normal person has changed since the technologisation of everyday life has led the normalisation of those who were formerly seen as possessing an excessive interest in machinery. We may find that what counts as ‘autismproper’ will be become further exaggerated from the ‘hypothetical normal person’, in contrast to these more useful autistic-like people. This is apparent in the recent search for the Broad Autism Phenotype. It changes the meaning of autism, but it doesn’t tell us anything useful about what it is to be autistic or how to support autistic individuals. Without recognising and representing the experiences of the neurodivergent in research design and implementation. Only aimed at understanding their ‘conditions’, we will simply reinforce the ‘normal/abnormal’ binary according to arbitrary new parameters.

Based on the third perspective, we are interested in drawing attention to the cultural construction of normality, particularly in relation to the mind. In this way, to be neurodivergent simply means to be outside of the category of people who are considered cognitive ‘normal’. It is a way of being a person that comes from institutional practices of diagnosis and medicalisation but it also involves the production of knowledge about what it means to be particular kind of person. To be neurodivergent is to reclaim the pathologizing aspects of a long-term cognitive diagnosis and to reclaim one’s neuro-status as a possible position from which to claim resources, representation and recognition.

From this perspective, we can begin to address the social construction of neurological difference, and the privileging of certain kinds of minds according to dominant ideologies. For instance, autism is associated with relatively less stigma than other forms of neurodivergence, not only in connection to supposed IT proficiency in its ‘milder forms’, but also with regards supposed affinities with nature.

The idea of a ‘neurodivergent’ identity is problematic where it assumes that there is a common experience of neurodiversity. It may even be used as a tool of oppression when those who are lacking in diagnosis or the correct configuration of traits based on what has gained cultural prominence at any moment in time. This is because who or what counts as neurodivergent will, depend on underlying neurology and physiology, along with broader cultural norms about what is appropriate behaviour, along with context and familiarity with particular diagnosed individuals. In this way it is highly individual and biosocial, even as we may gain through positively identifying with ‘non-normativity’. It is possible that at some point in time we no longer need the concept, as there is greater recognition of interdependence and social/environmental embeddedness.

If there is confusion over the ontological status of neurodiversity, it must result from the complex ‘engines of discovery’ where it is used both a concept of medical origins and something that is predicated of oneself in the light of self-knowledge. This suggest that what is needed is a reframing of the medical model so that it no longer necessarily assumes a disordered selfhood. Neurodiversity needs to be connected to theories of justice. As a result of this, clinicians would need to acknowledge epistemic violence that has been committed in the name of supporting neurodivergent people in the past. To reframe the medical model of neurodivergent conditions, we need to change the ways in which knowledge is produced. This means changing the epistemic norms. What we are asking for is a new kind of objectivity, which goes further to connect ‘insider’ and ‘outsider’ perspectives on self-knowledge. It may turn out that what we thought of as authoritative knowledge about other people based on observable behaviour may be nothing of the sort – that our own introspective capacities offer the greatest opportunity for discovery. Or yet, that neither introspection nor behaviour offer a reliable guide to ‘the bigger picture’ but that the bigger picture becomes clear as we recognise the limitation of our own individual positions.

While we must start with the categories defined by clinicians, we can move towards self representations. If we are able to acknowledge our own subjectivity – as the neurodivergent are so often required to do – we may begin the process of negotiating representation for many different neurotypes. We don’t yet know what epistemic or methodological rules will help us to get here. We commit to working across neurotypes, working with rather than ‘on’ other people. This means decolonializing neurodivergence/autism research and the hierarchies between neurotypes. This working with is therefore not the same as researching on or for, but also not the same as the “with” in which the neurodivergent becomes a strawman in an otherwise neurotypical led and defined research (Woods et al, 2018) This means recognising and questioning colonializing pasts and practices within research and practice, formulating other perspectives on knowledge and knowledge production and challenging dominant perspectives on research ethics. There are many examples of this in our edited book and elsewhere. We hope that this will contribute to the broader project of centralizing marginality, marginalising the centre which has been the project of feminist and postcolonial research for the past decades and which is also central to disability studies.
